# Deficits in Approximate Number System Acuity and Mathematical Abilities in 6.5-Year-Old Children Born Extremely Preterm

**DOI:** 10.3389/fpsyg.2017.01175

**Published:** 2017-07-11

**Authors:** Melissa E. Libertus, Lea Forsman, Ulrika Adén, Kerstin Hellgren

**Affiliations:** ^1^Department of Psychology, Learning Research and Development Center, University of Pittsburgh Pittsburgh, PA, United States; ^2^Department of Women’s and Children’s Health, Karolinska Institutet Stockholm, Sweden; ^3^Department of Clinical Neuroscience, Karolinska Institutet Stockholm, Sweden

**Keywords:** approximate number system, math ability, preterm children, extreme preterm birth, visuo-spatial skills, attention

## Abstract

Preterm children are at increased risk for poor academic achievement, especially in math. In the present study, we examined whether preterm children differ from term-born children in their intuitive sense of number that relies on an unlearned, approximate number system (ANS) and whether there is a link between preterm children’s ANS acuity and their math abilities. To this end, 6.5-year-old extremely preterm (i.e., <27 weeks gestation, *n* = 82) and term-born children (*n* = 89) completed a non-symbolic number comparison (ANS acuity) task and a standardized math test. We found that extremely preterm children had significantly lower ANS acuity than term-born children and that these differences could not be fully explained by differences in verbal IQ, perceptual reasoning skills, working memory, or attention. Differences in ANS acuity persisted even when demands on visuo-spatial skills and attention were reduced in the ANS task. Finally, we found that ANS acuity and math ability are linked in extremely preterm children, similar to previous results from term-born children. These results suggest that deficits in the ANS may be at least partly responsible for the deficits in math abilities often observed in extremely preterm children.

## Introduction

Math is important. Math skills at school entry are the best predictor of later academic success (Duncan et al., 2007), and adults’ math skills are critical for career success and mental and physical health (Parsons and Bynner, 2005; Reyna and Brainerd, 2007). However, children and adults differ dramatically in their academic achievement in general, and particularly in their math skills. Preterm children are at heightened risk for low academic achievement (Wocadlo and Rieger, 2007; Johnson et al., 2009, 2011) and especially low mathematical abilities (Pritchard et al., 2009; Taylor et al., 2009; Simms et al., 2013a). A meta-analysis, for example, found very preterm children (i.e., in this particular analysis those children born before or at 33 weeks of gestation) to score 0.6 SD below term-born peers in math and 0.48 SD below term-born peers in reading (Aarnoudse-Moens et al., 2009).

Math deficits in preterm children may be due to anomalous gray matter volume in parietal cortex (Isaacs et al., 2001), a part of the brain that is heavily implicated in solving math problems (Grabner et al., 2007; Ansari, 2008; Amalric and Dehaene, 2016). If math deficits are due to volumetric reductions in parietal cortex, then basic number processes that also rely on parietal cortex, may be affected by preterm birth as well. A study by Simms et al. (2013b) demonstrated the link between basic number processes and general mathematical ability in extremely preterm children. They showed that at 11 years of age children born extremely preterm scored significantly lower than their term-born peers on the Mathematics Estimation Test (MET). The MET required them to verbally estimate line lengths, the location of a number on a number line, the number of dots in an image, and the distance between two locations on a map. Importantly, these children’s performance on the MET was correlated with their performance on a standardized assessment of mathematics assessing a broad range of age-appropriate math concepts.

At an even more basic level, 6.5-year-old extremely preterm children (i.e., those born at less than 27 weeks gestation; Fellman et al., 2009) also perform significantly worse than their term-born peers on a non-symbolic number comparison task in which they had to compare two briefly flashed arrays of dots and state which one was more numerous (Hellgren et al., 2013). Because this non-symbolic number comparison task does not permit children to count the dots, it relies on an intuitive sense of number, also called the approximate number system (ANS; Dehaene, 1997; Halberda et al., 2008). The ANS is thought to be present from birth (Izard et al., 2009), functional in highly educated and uneducated people (Pica et al., 2004; Nys et al., 2013), and non-human animals (Cantlon and Brannon, 2006; Agrillo et al., 2008) and hence independent of language. Similar to math, the ANS relies on regions of parietal cortex (Nieder and Dehaene, 2009) and disruptions to the functioning of this part of the brain interfere with performance on simple number comparisons (Cappelletti et al., 2007; Dormal et al., 2008). Thus, one possible explanation for preterm children’s low performance in math and poorer acuity of the ANS may be that growth of the parietal cortex is particularly vulnerable when children are born prematurely (Padilla et al., 2015).

Some correlational studies found that term-born children with greater ANS acuity tend to perform better on standardized math tests (Halberda et al., 2008; Inglis et al., 2011; Libertus et al., 2011; Mazzocco et al., 2011a; Mussolin et al., 2012; Anobile et al., 2013; Bonny and Lourenco, 2013; Pinheiro-Chagas et al., 2014; vanMarle et al., 2014; Keller and Libertus, 2015) and earlier ANS acuity predicts later math abilities (Mazzocco et al., 2011b; Libertus et al., 2013a,b; Starr et al., 2013), while others fail to find such a link (Soltesz et al., 2010; Fuhs and McNeil, 2013; Gilmore et al., 2013; Sasanguie et al., 2014). Possible explanations for these mixed results are methodological issues assessing the ANS including the possibility that these tasks may tap into perceptual processing or inhibitory control rather than number processing (De Smedt et al., 2013; Dietrich et al., 2015; Gebuis et al., 2016; Leibovich and Ansari, 2016; Leibovich et al., 2016; Reynvoet and Sasanguie, 2016), variations in age (Inglis et al., 2011), or which aspects of math are assessed (Libertus et al., 2013b). However, recent meta-analyses have shown a small, but significant link between the ANS and math skills (Chen and Li, 2014; Fazio et al., 2014; Schneider et al., 2016). Further support for a link between the ANS and math comes from training studies. Improving ANS acuity through targeted training procedures has been shown to lead to subsequent improvements in math abilities (Park and Brannon, 2013, 2014; Hyde et al., 2014; Park et al., 2016; Wang et al., 2016). However, the validity of these studies has been questioned and their conclusions should be considered carefully (Lindskog and Winman, 2016; Merkley et al., 2017; but see Park and Brannon, 2016; Wang et al., 2017, for responses). Regardless of these debates in the literature, it is unclear whether there is a link between the ANS and math skills in preterm children.

Simms et al. (2015) examined several basic number processes in isolation and found no significant differences between preterm and term-born children’s performance on non-symbolic or symbolic (i.e., Arabic numeral) comparison tasks. They found differences on counting skills and arithmetic strategies, but preterm children’s deficits on these tasks could be explained by general deficits in working memory and visuo-spatial skills. Solving math problems relies heavily on working memory, e.g., when keeping intermediate results in mind while solving a math problem in one’s head, and visuo-spatial skills, e.g., when properly aligning thousands, hundreds, tens, and single units in a written arithmetic problem. Thus, there is ample evidence that individual differences in working memory and visuo-spatial skills correlate with differences in math abilities even in term-born children and adults (Espy et al., 2004; Gathercole et al., 2004).

Additionally, preterm birth is associated with deficits in a host of general cognitive factors including attention, working memory, inhibitory control, verbal IQ, and perceptual reasoning skills (Johnson, 2007; Aarnoudse-Moens et al., 2009; Mulder et al., 2009). For example, preterm birth increases the risk of attention-deficit hyperactivity disorder (ADHD) with increasing degree of prematurity leading to a greater likelihood of ADHD (Lindström et al., 2011). In term-born children, attention, working memory, and inhibitory control skills have been linked to math skills and ANS acuity (Espy et al., 2004; Blair and Razza, 2007; Gilmore et al., 2013). For example, 6- to 8-year-old children’s ability to suppress a prepotent response and flexibly switch between different learned rules uniquely predict their math skills (Bull and Scerif, 2001). Similarly, variability in the need to inhibit irrelevant perceptual information during a non-symbolic number comparison task is associated with variability in ANS acuity (Szucs et al., 2013). In addition, term-born children’s language abilities and perceptual reasoning skills have been associated with their math skills (Blair and Razza, 2007; LeFevre et al., 2010). Thus, it is possible that preterm children’s deficits in math and ANS acuity could be explained by underlying deficits in attention, working memory, inhibitory control, verbal IQ, and perceptual reasoning skills.

In sum, the goals of the present study were four-fold: First, we sought to replicate the differences in ANS acuity in a larger sample of extremely preterm and term-born children (Hellgren et al., 2013). Second, we wanted to assess whether differences in the demands on visuo-spatial skills – measured during the ANS acuity task – could explain ANS acuity differences between extremely preterm and term-born children. Third, we wanted to assess whether differences in attentional demands and inhibitory control – again measured during the ANS acuity task – could explain ANS acuity differences between extremely preterm and term-born children. Finally, we sought to determine the relation between extremely preterm children’s ANS acuity and their math ability. To this end, 6.5-year-old extremely preterm (i.e., <27 weeks gestation) and term-born children completed a non-symbolic number comparison task (ANS acuity task) comprised of trials with lesser or greater demands on visuo-spatial skills (i.e., trials in which dots were spatially separated or spatially intermixed) and trials in which inhibitory control demands were lesser or greater (i.e., trials in which the cumulative surface area of all dots was greater for the more numerous array or trials in which surface area was equated between both arrays). Children also completed a standardized math test. Finally, to control for potential individual differences in general cognitive skills, children completed standardized assessments designed to measure working memory, verbal IQ and perceptual reasoning skills and parents completed a questionnaire rating their children’s attention skills.

## Materials and Methods

### Participants

The participants in the current study represent the Stockholm cohort of a national, population-based study of extremely preterm born (EP) children in Sweden (Fellman et al., 2009). The national study was initiated to investigate the mortality and long-term morbidity of children born at gestational age (GA) < 27 weeks. Neurodevelopmental and ophthalmologic outcomes at 6.5 years have been presented elsewhere (Hellgren et al., 2016; Serenius et al., 2016). ANS data from a subset of the participants (*N* = 86, 43 extremely preterm born and 43 term-born children) in the current study have been presented previously (Hellgren et al., 2013).

The EP group had been recruited at birth and, in the present study, comprised all surviving infants born before GA 27 weeks in the Stockholm area between January 1st, 2004 and March 31st, 2007. There were 120 eligible EP children who were invited to the current study at 6.5 years of age together with a matched comparison group of term-born (TB) children. The TB children were matched for age (uncorrected to gestation at birth), sex and home zip code to the EP children. Eighty-two EP children (38 girls, i.e., 46%) and 89 TB children (36 females, i.e., 40%) completed the tasks. An additional 38 EP children (9 females, i.e., 24%) and 7 TB children (4 females, i.e., 57%) did not contribute data because of a lack of availability to complete the current testing wave (EP: *n* = 21; TB: *n* = 3) or an inability to complete the assessments included in the current report (EP: *n* = 17; TB: *n* = 4). Of those children who were unable to complete the assessments included in the current report, there were three EP children who were blind and twelve EP children who had cognitive deficits. One TB child was visually impaired and autistic. Finally, two EP and three TB children did not contribute data because of technical difficulties during testing. The 38 EP children who did not contribute data had significantly lower birth weight (*M* = 772 g; *SD* = 161 g) and lower GA (*M* = 25.1 weeks; *SD* = 1.1 weeks) at birth than the 82 participants who contributed data [birth weight: *M* = 834 g; *SD* = 152 g, *t*(118) = -2.02; *p* = 0.045, Cohen’s *d* = 0.4; GA: *M* = 25.5 weeks; *SD* = 0.9 weeks, *t*(61.24) = -2.09; *p* = 0.04, Cohen’s *d* = 0.4]. The mean birth weight and GA of the 89 participating TB children were 3653 g (*SD* = 432 g) and 39.9 weeks (*SD* = 1.2 weeks), respectively.

The regional ethics committee in Stockholm approved the study and written informed consent was obtained from all parents of the participating children before testing. Children provided verbal assent prior to participating.

### Materials and Procedure

#### Approximate Number System (ANS) Acuity Task

To measure children’s ANS acuity, they completed Panamath^1^, a freely available, non-symbolic number comparison task that has been widely used in previous studies to assess children’s and adults’ ANS acuity (Halberda et al., 2008; Libertus et al., 2011, 2013a,b; Hellgren et al., 2013; Keller and Libertus, 2015). Children sat at a table approximately 60 cm away from a 19″ computer screen and were shown two arrays of blue and yellow dots simultaneously displayed on the screen for 2000 ms followed by a blank screen until a response was made. Children were asked to say which array was more numerous by naming the appropriate color and the experimenter entered the response by pressing one of two keys on the keyboard. While no formal comparison has been made between RTs derived from verbal and button press responses in ANS acuity tasks, pilot testing has shown that many children struggle with learning to press a button corresponding to their answer and find naming a color easier. In addition, previous research has shown that extreme prematurity is associated with deficits in motor skills at 6 years of age (Marlow et al., 2007). Thus, to ensure that difficulties in learning the correct button responses could not explain our findings and in keeping with previously published papers (Libertus et al., 2011, 2013a,b; Keller and Libertus, 2015; Braham and Libertus, 2016), we decided to have the experimenter press a button for the child.

Children were first presented with practice trials until they understood the task. Then they completed 48 test trials. For half of the trials, there were more blue than yellow dots, and for the other half of the trials, there were more yellow than blue dots. Children were instructed to respond as quickly and as accurately as possible and received no feedback about the correctness of their response.

**FIGURE 1:**
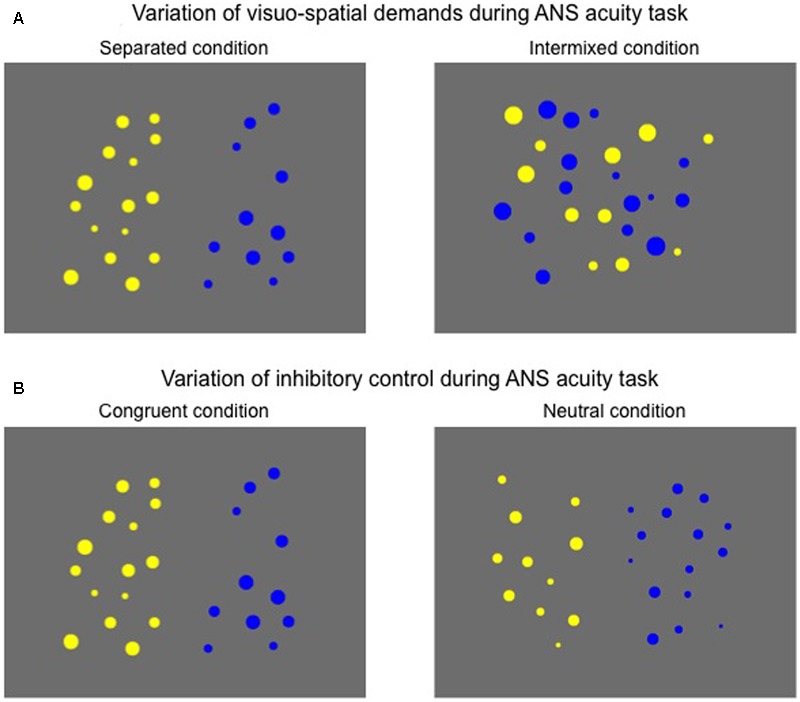
Sample stimuli used in the ANS acuity task. **(A)** For half of the trials, blue and yellow dots were presented spatially separated on the left and the right side of the screen (Separated condition), and for the other half of the test trials, blue and yellow dots were presented intermixed at the center of the screen (Intermixed condition). **(B)** Orthogonally, half of the trials contained arrays of blue and yellow dots with the same average dot size, i.e., the more numerous array had the larger cumulative surface area (Congruent condition). The other half of the trials contained arrays of blue and yellow dots that were equated for cumulative surface area (Neutral condition).

Each array contained between 5 and 16 dots, varying in dot size (average dot size = 46 pixels, *SD* = 10 pixels). For half of the trials, blue and yellow dots were presented spatially separated on the left and the right side of the screen (Separated condition; see **Figure 1A**), and for the other half of the test trials, blue and yellow dots were presented intermixed at the center of the screen (Intermixed condition). Orthogonal to the Separate and Intermixed conditions, half of the trials contained arrays of blue and yellow dots with the same average dot size, i.e., the more numerous array had the larger cumulative surface area (Congruent trials; see **Figure 1B**). The other half of the trials contained arrays of blue and yellow dots that were equated for cumulative surface area (Neutral trials). Thus, Neutral trials eliminate cumulative surface area as a perceptual cue that is often confounded with number. Importantly, while Panamath and other non-symbolic number comparison tasks have often been criticized for their failure to control for convex hull (Gebuis and Reynvoet, 2012; Clayton et al., 2015; Gilmore et al., 2016), the ratio between the numerosities in each dot array is a significant predictor of children’s and adults’ performance on non-symbolic number comparison tasks in which stimuli are generated using the Panamath software even when controlling for the ratio between the arrays’ convex hulls (Libertus et al., in preparation).

Trial difficulty was varied by using different ratios between the two arrays of dots. Ratio was counterbalanced across the Separated and Intermixed condition as well as the Congruent and Neutral trials. For the first 88 participants (45 EP children and 43 TB children), there were three different ratios: 2.3 (e.g., 14 yellow dots and 6 blue dots), 1.7 (e.g., 12 yellow dots and 7 blue dots), and 1.3 (e.g., 13 yellow dots and 10 blue dots). For the remaining 89 participants (42 EP children and 47 TB children), we added a fourth, more difficult ratio, i.e., 1.15 (e.g., 15 yellow dots and 13 blue dots) because many children in the TB group performed close to ceiling when using only three ratios. The number of test trials was identical in the two versions because we reduced the number of trials per ratio when adding in the fourth ratio.

#### Verbal IQ

Verbal IQ was measured with the Wechsler Intelligence Scale for Children IV (WISC-IV; Wechsler, 2003). The Verbal IQ score was based on the combined scaled scores on the Similarities, Vocabulary, and Comprehension subtests.

#### Perceptual Reasoning Skills

Perceptual reasoning skills were measured with the Block Design subtest of the WISC-IV (Wechsler, 2003). Scaled scores were used as the dependent measure.

#### Working Memory

Working memory skills were measured with the Digit Span subtest of the WISC-IV (Wechsler, 2003). Scaled scores were used as the dependent measure.

#### Attention

The Attention scale of the Brown Attention Deficit Disorder Scales (BADDS; Brown, 2001) was used to measure attention. For children between 3 and 7 years of age, the BADDS consists of a 40-item parent and teacher report measure designed to assess a wide range of executive functions. The Attention scale is comprised of eight questions about the child’s ability to focus, sustain and shift attention in relation to various tasks and the rater selects the answer on a scale of zero to three, where 0 = Never and 3 = Almost Daily. In this study, the child’s parent or guardian completed the BADDS. We used raw scores on the BADDS Attention scale as the dependent measure for children’s attention skills.

#### Math Ability

The Arithmetic subtest from the WISC-IV (Wechsler, 2003) was used to measure general mathematical ability. This subtest consists of a series of orally administered mathematical problems that must be solved without pen and pencil. The problems are similar to problems that would be encountered in an elementary math class and often are presented in a story format. The Arithmetic subtest was only added to the testing battery after approximately the first half of the study; hence, only 54 EP and 51 TB children completed this assessment. We used raw scores on the WISC-IV Arithmetic subtest as the dependent measure for children’s math ability.

### Data Analyses

#### Approximate Number System (ANS) Acuity Task

We calculated children’s accuracy as the percentage of correct test trials, their average response time across all trials, and their Weber fractions (*w*). To determine each individual child’s *w*, we fit each child’s responses over all 48 trials with a widely used psychophysical model (1) (cf., Green and Swets, 1966; Pica et al., 2004; Halberda and Feigenson, 2008; Halberda et al., 2008).

(1) expected accuracy=Φ(ratio−1wratio2+1)

In this model, *ratio* is the ratio between the presented numerosities (larger number/smaller number), *w* the Weber fraction, and Φ the standard cumulative distribution function of a Gaussian distribution. The best-fitting *w* parameter was found via non-linear least squares. The model assumes that the underlying approximate number representations are distributed along a continuum of Gaussian random variables. An important implication of this model is that the two numbers of dots presented on each trial will often yield similar and overlapping representations. In other words, as the ratio of two numerosities becomes increasingly close (i.e., approaches 1.0), their Gaussian representations will increasingly overlap and children will have greater difficulty determining which array is more numerous, resulting in decreased accuracy. A smaller *w* indicates greater acuity in a child’s ANS representations. Data from five EP children and one TB child could not be fit using the model because the children performed at chance across all ratios.

To assess preliminary group differences between EP and TB children’s ANS acuity, we calculated a MANOVA with Group (EP vs. TB) as a between-subject factor and accuracy, RT, and *w* as dependent variables. As expected, we found a significant effect of Group, *F*(3,167) = 11.00, *p* < 0.001, $\eta_{p}^{2}$ = 0.17. Univariate analyses of variance for each dependent variable revealed significant effects of Group for all three measures of ANS acuity [accuracy: *F*(1,169) = 16.99, *p* < 0.001, $\eta_{p}^{2}$ = 0.09; RT: *F*(1,169) = 14.29, *p* < 0.001, $\eta_{p}^{2}$ = 0.08; *w*: *F*(1,169) = 20.26, *p* < 0.001, $\eta_{p}^{2}$ = 0.11]. Next, to determine whether these group differences persist when controlling for the two different versions of the ANS acuity task (3 vs. 4 different ratios), we calculated a MANCOVA with Group as a between-subject factor, accuracy, RT and *w* as dependent variables and Task Version as a covariate. As before, we found a significant effect of Group, *F*(3,166) = 11.00, *p* < 0.001, $\eta_{p}^{2}$ = 0.17. Task Version was a significant covariate, *F*(3,166) = 37.84, *p* < 0.001, $\eta_{p}^{2}$ = 0.41. Follow-up ANCOVAs for each dependent variable revealed significant effects of Group for all three measures of ANS acuity even when controlling for Task Version [accuracy: *F*(1,168) = 21.99, *p* < 0.001, $\eta_{p}^{2}$ = 0.12; RT: *F*(1,168) = 13.71, *p* < 0.001, $\eta_{p}^{2}$ = 0.08; *w*: *F*(1,168) = 20.89, *p* < 0.001, $\eta_{p}^{2}$ = 0.11]. For accuracy and RT, Task Version was a significant covariate [accuracy: *F*(1,168) = 26.73, *p* < 0.001; RT: *F*(1,168) = 5.58, *p* = 0.02]. Task Version was not a significant covariate for *w, F*(1,168) = 1.66, *p* = 0.20.

In keeping with previously published work (Hellgren et al., 2013) and to simplify our main analyses, we combined RT and *w* into a single measure of ANS acuity by first computing separate *z*-scores for RT and *w* based on the means and standard deviations of the TB group and then averaging the *z*-scores for RT and *w* for each child. A lower value on this combined ANS acuity measure indicates greater ANS acuity. Combined scores of accuracy-based and RT-based measures of ANS acuity have been used in the past (Simon et al., 2008; Sasanguie et al., 2012a,b; Bartelet et al., 2014) and it has been suggested that they are particularly appropriate when comparing performance in two groups of children where one is expected to make more errors than the other (Bish et al., 2005; Simon et al., 2008). However, instead of using the more common combined measure of inverse efficiency (RT/proportion of correct trials), we used the *z*-score based average described above. Recent work has shown that calculating a combined score in the way described here captures variance in the accuracy-based and RT-based measure equally (Dietrich et al., 2016). Thus, it has been proposed that this combined measure can be interchangeably used with accuracy-based and RT-based measures (Dietrich et al., 2016).

To determine whether the two different versions of the ANS acuity task affected this combined measure of ANS acuity, we calculated a one-way ANOVA with Task Version (3-ratio vs. 4-ratio) as a between-subject factor. There was no significant main effect of Task Version, *F*(1,169) = 2.04, *p* = 0.16, $\eta_{p}^{2}$ = 0.01. Hence, for all analyses including the combined ANS acuity measure, we collapsed across the two different task versions of the ANS acuity task.

### Statistical Analyses

First, we assessed whether preterm and term-born children differed in ANS acuity and whether such differences could be explained by differences in verbal IQ, perceptual reasoning skills as measured via the Block Design subtest on the WISC-IV, working memory as measured via the Digit Span subtest on the WISC-IV, and attention skills as measured with the Attention scale on the BADDS using analyses of variance (ANOVAs). Second, to determine whether variations in visuo-spatial features of the stimuli during the ANS task may explain differences in ANS acuity, we compared children’s performance on the Separated and Intermixed conditions of the ANS acuity task using ANOVAs. Third, to determine whether attentional and inhibitory control demands during the ANS task explained differences in ANS acuity, we compared children’s performance on the Congruent and Neutral trials of the ANS acuity task using ANOVAs. Finally, we examined whether individual differences in preterm children’s ANS acuity are related to their math ability on a standardized math assessment and whether this relation could be explained by individual differences in verbal IQ, perceptual reasoning skills, working memory as well as attention using regression analyses.

## Results

Descriptive results of all measures are presented in **Table 1**.

**Table 1:** Descriptive results of ANS acuity, verbal IQ, perceptual reasoning skills (Block Design subtest on WISC-IV), working memory (Digit Span subtest on WISC-IV), attention (BADDS Attention subscale), and math ability (Arithmetic subtest on the WISC-IV) for extremely preterm (EP) and term-born (TB) children, respectively.

	EP group	TB group
Mean ANS acuity (SD)	1.16 (1.83)	0.004 (0.72)
Mean verbal IQ (SD)	27.36 (8.03)	35.44 (5.13)
Mean perceptual reasoning (SD)	9.69 (2.73)	11.78 (2.47)
Mean working memory (SD)	6.40 (2.51)	8.29 (2.10)
Mean attention score (SD)	7.30 (5.91)	3.92 (3.49)
Mean arithmetic score (SD)	9.48 (3.97)	13.28 (2.82)

### Differences in ANS Acuity between Preterm and Term-Born Children

To determine whether preterm and term-born children differed in ANS acuity, we calculated a univariate ANOVA with Group (EP vs. TB) as a between-subject factor and the combined ANS acuity measure as the dependent variable. We found a significant main effect of Group, *F*(1,169) = 30.67, *p* < 0.001, $\eta_{p}^{2}$ = 0.15, which was due to a more precise ANS in the TB group than the EP group. These results remained significant even when controlling for Task Version (3-ratio vs. 4-ratio), *F*(1,168) = 29.66, *p* < 0.001, $\eta_{p}^{2}$ = 0.15.

To assess whether the differences in ANS acuity between preterm and term-born children can be explained by differences in verbal IQ, perceptual reasoning skills, working memory, and/or attention, we calculated the same univariate ANOVA as before but added the verbal IQ, the Block Design and Digit Span subtest scores from the WISC-IV and the attention score from the BADDS as covariates to the model. Again, we found a significant effect of Group, *F*(1,159) = 9.13, *p* < 0.01, $\eta_{p}^{2}$ = 0.05, even when controlling for verbal IQ, perceptual reasoning skills, working memory, and attention. These results remained significant even when additionally controlling for Task Version, *F*(1,158) = 4.01, *p* < 0.05, $\eta_{p}^{2}$ = 0.03. Finally, to check whether these group differences in ANS acuity are carried primarily by RT or *w*, we ran two separate univariate ANOVAs controlling for verbal IQ, perceptual reasoning skills, working memory, attention, and task version. For RT, we found a significant effect of Group, *F*(1,163) = 5.59, *p* < 0.02, $\eta_{p}^{2}$ = 0.03. For *w*, the main effect of Group was marginally significant, *F*(1,158) = 3.14, *p* = 0.08, $\eta_{p}^{2}$ = 0.02, suggesting that RT contributes more heavily to the observed differences between preterm and term-born children’s ANS acuity, but that *w* shows a similar pattern.

### Can Visuo-spatial Skills Explain ANS Differences between Preterm and Term-Born Children?

Even though the previous analyses showed that group differences in ANS acuity persist when controlling for perceptual reasoning skills, we conducted a complementary analysis to determine whether visuo-spatial aspects of the ANS acuity task may explain the observed differences between preterm and term-born children. To this end, we compared children’s performance on the Separated and Intermixed conditions of the ANS acuity task, i.e., trials in which blue and yellow dots were spatially separated on the left and right side of the screen and trials in which blue and yellow dots were spatially intermixed in the center of the screen. Because there are only 24 trials in each of the two conditions, we were unable to calculate *w* for each condition separately. Instead, we used accuracy and RT as measures of ANS acuity in each condition and added Task Version (3-ratio vs. 4-ratio) as a covariate to our analyses to control for differences between the two versions of the ANS acuity task.

A mixed-design ANOVA on *accuracy* with Condition (Separated vs. Intermixed) as a within-subject factor and Group (EP vs. TB) and Task Version (3-ratio vs. 4-ratio) as between-subject factors revealed a significant main effect of Group, *F*(1,173) = 24.46, *p* < 0.001, $\eta_{p}^{2}$ = 0.12, and a significant main effect of Task Version, *F*(1,173) = 16.56, *p* < 0.001, $\eta_{p}^{2}$ = 0.09, but no main effect of Condition, *F*(1,173) = 0.99, *p* = 0.32, $\eta_{p}^{2}$ = 0.006. There was also a significant interaction between Condition and Task Version, *F*(1,173) = 5.69, *p* = 0.02, $\eta_{p}^{2}$ = 0.03, but no interaction between Group and Task Version, *F*(1,173) < 0.01, *p* = 0.94, $\eta_{p}^{2}$ < 0.001. Critically, there was no significant interaction between Group and Condition, *F*(1,173) = 0.90, *p* = 0.34, $\eta_{p}^{2}$ = 0.005, or between Group, Condition, and Task Version, *F*(1,173) = 1.30, *p* = 0.26, $\eta_{p}^{2}$ = 0.007. These results suggest that visuo-spatial aspects of the ANS task do not affect EP and TB children’s ANS accuracy differentially.

We calculated a parallel mixed-design ANOVA on *RT* and found a significant main effect of Condition, *F*(1,173) = 3.95, *p* < 0.05, $\eta_{p}^{2}$ = 0.02, a significant main effect of Group, *F*(1,173) = 16.75, *p* < 0.001, $\eta_{p}^{2}$ = 0.09, and a significant main effect of Task Version, *F*(1,173) = 5.61, *p* < 0.05, $\eta_{p}^{2}$ = 0.03. There was also a significant interaction between Group and Task Version, *F*(1,173) = 4.45, *p* < 0.05, $\eta_{p}^{2}$ = 0.03, but no interaction between Condition and Task Version, *F*(1,173) = 0.04, *p* = 0.85, $\eta_{p}^{2}$ < 0.001. Most importantly, there were no significant interactions between Group and Condition, *F*(1,173) = 0.07, *p* = 0.80, $\eta_{p}^{2}$ < 0.001, or between Group, Condition, and Task Version, *F*(1,173) = 0.02, *p* = 0.88, $\eta_{p}^{2}$ < 0.001. Similar to our accuracy results, these results suggest that visuo-spatial aspects of the ANS task do not affect EP and TB children’s ANS RT differentially.

### Can Attentional Demands Explain ANS Differences between Preterm and Term-Born Children?

Even though our initial analyses showed that group differences in ANS acuity persist when controlling for attention skills as measured by the Attention scale on the BADDS, we conducted a complementary analysis to determine whether variations in the need to attend to different stimulus dimensions during the ANS acuity task may explain the observed differences between preterm and term-born children. To this end, we compared children’s performance on the Congruent and Neutral trials of the ANS acuity task. For Congruent trials, the more numerous array also had the larger cumulative surface area, while for Neutral trials, both arrays had equal cumulative surface area. Thus, Congruent trials provided children with multiple cues to determine the correct answer and hence a lesser demand on their attention (Fuhs and McNeil, 2013; Gilmore et al., 2013). Similar to our analyses above and because there are only 24 trials in each of the two trial types, we were unable to calculate *w* for each trial type separately. Instead, we used accuracy and RT as measures of ANS acuity and added Task Version (3-ratio vs. 4-ratio) as a covariate to our analyses to control for differences between the two versions of the ANS acuity task.

A mixed-design ANOVA on *accuracy* with Trial Type (Congruent vs. Neutral) as a within-subject factor and Group (EP vs. TB) and Task Version (3-ratio vs. 4-ratio) as between-subject factors revealed significant main effects of Trial Type, *F*(1,173) = 127.20, *p* < 0.001, $\eta_{p}^{2}$ = 0.42, Group, *F*(1,173) = 23.84, *p* < 0.001, $\eta_{p}^{2}$ = 0.12, and Task Version, *F*(1,173) = 17.51, *p* < 0.001, $\eta_{p}^{2}$ = 0.09. There was also a significant interaction between Trial Type and Task Version, *F*(1,173) = 25.96, *p* < 0.001, $\eta_{p}^{2}$ = 0.13, but no interaction between Group and Task Version, *F*(1,173) = 0.03, *p* = 0.87, $\eta_{p}^{2}$ < 0.001. Critically, there was no significant interaction between Group and Trial Type, *F*(1,173) = 1.68, *p* = 0.20, $\eta_{p}^{2}$ = 0.01, or between Group, Trial Type, and Task Version, *F*(1,173) = 0.23, *p* = 0.63, $\eta_{p}^{2}$ = 0.001. These results suggest that differences in attentional demands during the ANS task do not affect EP and TB children’s ANS accuracy differentially.

We calculated a parallel mixed-design ANOVA on *RT* and found significant main effects of Trial Type, *F*(1,173) = 16.48, *p* < 0.001, $\eta_{p}^{2}$ = 0.09, Group, *F*(1,173) = 17.99, *p* < 0.001, $\eta_{p}^{2}$ = 0.09, and Task Version, *F*(1,173) = 5.59, *p* < 0.05, $\eta_{p}^{2}$ = 0.03. There was also a significant interaction between Group and Task Version, *F*(1,173) = 4.35, *p* < 0.05, $\eta_{p}^{2}$ = 0.02, as well as a significant interaction between Trial Type and Task Version, *F*(1,173) = 5.84, *p* < 0.02, $\eta_{p}^{2}$ = 0.03. Most importantly, there were no significant interactions between Group and Trial Type, *F*(1,173) = 2.48, *p* = 0.12, $\eta_{p}^{2}$ = 0.01, or between Group, Trial Type, and Task Version, *F*(1,173) = 0.44, *p* = 0.51, $\eta_{p}^{2}$ = 0.003. Similar to our accuracy results, these results suggest that differences in attentional demands during the ANS task do not affect EP and TB children’s ANS RT differentially.

### Relation between ANS Acuity and Math Ability in Preterm Children

Previous research suggests a link between ANS acuity and math ability in typically developing children (Libertus et al., 2011, 2013a,b; Bonny and Lourenco, 2013; Starr et al., 2013). Here, we examine whether individual differences in preterm children’s ANS acuity are related to their math ability on a standardized math assessment. Note that only 54 EP children completed the standardized math assessment because it was only added to the testing battery after approximately the first half of the study. We found a significant correlation between the combined measure of ANS acuity in preterm children and their performance on the Arithmetic subtest of the WISC-IV, *R* = -0.40, *p* < 0.01^2^. In line with previous results in typically developing children (Keller and Libertus, 2015) (but see Fuhs and McNeil, 2013; Gilmore et al., 2013, for opposing results), this relation also held when considering ANS acuity separately as accuracy on Congruent, *R* = 0.48, *p* < 0.001, and Neutral trials, *R* = 0.41, *p* = 0.001. Similarly, we found significant correlations between math and accuracy on the Separated condition of the ANS acuity task, *R* = 0.51, *p* < 0.001, and the Intermixed condition, *R* = 0.41, *p* = 0.001.

Next, we examined whether this relation between ANS acuity and math ability could be explained by individual differences in verbal IQ, perceptual reasoning skills as measured via the Block Design subtest on the WISC-IV, working memory as measured via the Digit Span subtest on the WISC-IV, and/or attention as measured by the BADDS. To this end, we conducted a hierarchical linear regression analysis with preterm children’s Arithmetic score as the dependent variable. In the first model (Model 1), we entered children’s verbal IQ, Block Design and Digit Span subtest scores on the WISC-IV as well as their attention score from the BADDS as potential predictors of their math ability. In the second model (Model 2), we added children’s combined ANS acuity score to determine whether ANS acuity predicted additional variance in math ability above and beyond verbal IQ, perceptual reasoning skills, working memory, and attention.

As can be seen in **Table 2**, Model 1 was highly significant and explained 68% of the variance in preterm children’s math scores. Verbal IQ, perceptual reasoning skills, and working memory were significant unique predictors, while attention scores did not explain any additional variance. Adding ANS acuity scores in Model 2 explained an additional 3% of variance in children’s math scores. ANS acuity was a marginally (*p =* 0.05) significant unique predictor above and beyond verbal IQ, perceptual reasoning skills, working memory, and attention.

**Table 2:** Regression models predicting preterm children’s math scores.

	Model 1		Model 2	
	*B*	*SE B*	*B*	*SE B*
Verbal IQ	0.14^∗^	0.06	0.14^∗^	0.06
Perceptual reasoning skills	0.51^∗∗^	0.15	0.45^∗^	0.15
Working memory	0.54^∗^	0.17	0.51^∗^	0.17
Attention	-0.03	0.07	-0.02	0.06
ANS acuity			-0.36^†^	0.18
*R*^2^	0.68^∗∗^		0.70^∗∗^	
*F*	25.93^∗∗^		22.82^∗∗^	
Change in *R*^2^			0.03^†^	
*F*_Change_			4.01^†^	

*B reflects the unstandardized coefficients for each variable.*

*^†^0.05, ^∗^*p* < 0.05, ^∗∗^*p* < 0.001.*

## Discussion

Our study resulted in four main findings: First, we extended the findings by Hellgren et al. (2013) showing differences in ANS acuity in a larger sample of extremely preterm and term-born children. Second, we showed that differences in the demands on visuo-spatial skills during the ANS acuity task cannot fully explain ANS acuity differences between extremely preterm and term-born children. Third, we showed that differences in attentional demands and inhibitory control during the ANS acuity task cannot fully explain ANS acuity differences between extremely preterm and term-born children. Finally, we found that ANS acuity and math ability are linked in extremely preterm children.

Children born extremely preterm showed significantly lower ANS acuity than their term-born peers even when controlling for verbal IQ, perceptual reasoning skills, working memory, and attention. When examining RT and *w* on the ANS acuity task in isolation, RT showed significant group differences while the results for *w* were only marginal. At first sight, these findings might suggest that the observed differences in performance on the ANS acuity task merely reflect deficits in general information processing previously associated with prematurity (Rose and Feldman, 1996). However, previous research has shown that RT on an ANS acuity task was a unique predictor of term-born children’s math ability even when controlling for accuracy on the task and general information processing speed as measured via RT on a computerized, non-numerical task (Libertus et al., 2013a). Thus, RT on an ANS acuity task taps into processes specific to the ANS and that are similarly related to math abilities as accuracy-based measures. Therefore, we believe that the performance differences between extremely preterm and term-born children on the ANS acuity task in the present study reflect – at least in part – important differences in ANS acuity. Future studies should include measures of information processing speed to examine the contribution of domain-general processing speed for this deficit in ANS acuity.

Even though preterm children often struggle with general visuo-spatial skills and inhibitory control (Marlow et al., 2007; Aarnoudse-Moens et al., 2009; Mulder et al., 2009), the differences in ANS acuity persisted regardless of the degree to which visuo-spatial skills and inhibitory control were needed during the non-symbolic number comparison tasks. Specifically, our results showed that group differences in ANS acuity were present regardless of the spatial arrangement of the dot arrays (i.e., spatially separated vs. intermixed) or the demands on inhibitory control and attention (i.e., total surface area of the dots congruent with number or equated between the two arrays). Thus, the differences in ANS acuity observed in our study cannot be explained by deficits in a variety of cognitive functions often impaired by prematurity. Instead our findings suggest that extreme prematurity leads to deficits in approximate number representations, which may result from impairments in parietal cortex functioning.

Our findings contradict those by Simms et al. (2015) who found no significant differences in ANS acuity between their samples of preterm and term-born 8- to 10-year-old children. Several differences between their sample and our sample may explain these divergent findings. First, we compared children at 6.5 years of age whereas Simms et al. (2015) tested children at a mean age of 9 years. It is possible that preterm children may be able to catch up over time and that by 9 years of age, the ANS acuity gap becomes negligible. Second, our sample of preterm children was born extremely prematurely (<27 weeks of gestation) whereas the sample by Simms et al. (2015) consisted of children born very preterm (<32 weeks of gestation). It is possible that an additional few weeks *in utero* can alter development significantly and lead to a lesser impact on foundational numerical skills such as the ANS. In fact, a recent study using functional magnetic resonance imaging of the fetal brain showed that the parietal lobe undergoes significant changes in functional connectivity to other brain regions between weeks 27 and 28 of gestation suggesting that extremely preterm children may be particularly susceptible to deficits in parietal cortex functioning (Jakab et al., 2014). Further evidence for this possible explanation comes from findings of impairments in parietal cortex assessed neonatally in the same cohort as in the present study (Padilla et al., 2015). Future studies need to carefully control the age at which preterm children are tested and the degree of prematurity to disentangle their respective effects on ANS acuity.

Extremely preterm children showed a similar link between ANS acuity and math ability as previously observed in term-born children (Libertus et al., 2011, 2013a; Bonny and Lourenco, 2013), suggesting that the ANS provides a foundation for learning math regardless of prematurity. However, the exact role of the ANS for mathematical ability is still unclear. There are four, not necessarily mutually exclusive possibilities. One possibility is that the ANS aids in the acquisition of exact number knowledge (Piazza, 2010; Wagner and Johnson, 2011; Gunderson et al., 2015). Children may use the ANS to learn the meaning of number words and children who have more precise ANS representations may be able to learn those meanings earlier and/or more easily. Earlier or faster acquisition of number words may lead to earlier or faster acquisition of subsequent math concepts resulting in the observed link between ANS acuity and general math ability. Support for this view comes from a correlational study showing that children’s counting skills and understanding of cardinality mediate the relation between the ANS and children’s math abilities (vanMarle et al., 2014).

A second possibility is that the mapping between number symbols (including number words as well as Arabic numerals) and the ANS is linked to math abilities (De Smedt and Gilmore, 2011; De Smedt et al., 2013). This hypothesis posits that a strong association between number symbols and their meaning (i.e., ANS representations) is associated with greater math abilities. Support for this view comes from studies showing that children’s number estimation performance (i.e., their ability to estimate the number of objects in a stimulus) mediates the link between ANS acuity and math abilities (Pinheiro-Chagas et al., 2014; Libertus et al., 2016). In contrast to the first hypothesis, the ANS would be linked to a variety of different number symbols, but the mapping may occur at a later point in time than during the initial acquisition of the number symbols.

A third possibility is that the ANS provides a sense of ordinality and directionality for arithmetic operations (McCrink et al., 2007; Knops et al., 2009; Lyons and Beilock, 2011). The ANS may support an understanding of the serial positioning of numbers along a mental number line and the notion that addition is associated with an increase in quantity and movement toward the right on the number line whereas subtraction is associated with a decrease in quantity and a movement toward the left. As such, the ANS could also be related to detecting errors when solving math problems as it may provide a general sense of the magnitude of the expected answer. Support for this hypothesis comes from a study showing that adults’ ability to detect numerically ordered sequences mediates the relation between their ANS acuity and math ability (Lyons and Beilock, 2011).

Finally, a fourth possibility is that the ANS and math ability are linked via a general attitude toward and ease with math (Braham and Libertus, under review). For example, adults with greater math anxiety perform worse on a symbolic number comparison task suggesting that math anxiety does not only affect performance on math assessments but also basic number processing tasks (Maloney et al., 2011). Moreover, parents with greater ANS acuity and greater math ability tend to talk more about numbers with their children (Elliott et al., 2017).

### Limitations and Future Directions

While we controlled for verbal IQ, perceptual reasoning skills, working memory, and attention in our analyses, future studies should increase the number of general cognitive abilities that are controlled and improve the way in which they are assessed. Because children already completed a large number of tests, attention skills were assessed via a broad parent-report measure. To assure construct validity, it would be useful to assess children’s attention skills directly in future studies. Additionally, we did not assess children’s inhibitory control directly. Since inhibitory control has been linked to math ability (Espy et al., 2004; Blair and Razza, 2007) and is affected by prematurity (Aarnoudse-Moens et al., 2009), a direct assessment (e.g., via a Go/No-go task) would be beneficial in the future. Also, working memory was tested via a digit span task, which may conflate deficits in working memory and math skills. Thus, future studies should use working memory tasks that do not use numbers as stimuli to properly control for working memory skills without tapping into processes related to math. Finally, response times in our ANS acuity task were recorded via a button response made by the experimenter as soon as the child gave a verbal response. Pilot testing showed that many children struggled with learning to press a button corresponding to their answer and found naming a color easier. However, the validity of these response times needs to be empirically tested.

Recent training studies showing that improvements in ANS acuity may transfer to improvements in math ability, hold promise for early interventions in preterm children. For example, 3- to 5-year-old typically developing, low-income children who were trained on a non-symbolic approximate arithmetic tasks daily for 2–3 weeks showed significantly greater math abilities after training than a group of children completing memory training (Park et al., 2016). Future studies could use a similar intervention to test whether it is effective in closing or at least decreasing the gaps in ANS acuity and math ability between extremely preterm and term-born children.

## Conclusion

In sum, our study showed that children born extremely preterm have significantly less precise approximate number representations and that these deficits cannot be fully explained by cognitive deficits in areas such as verbal IQ, perceptual reasoning skills, working memory, or attention. Moreover, we found an association between preterm children’s ANS acuity and their math abilities suggesting that deficits in the ANS may be at least in part responsible for the deficits in math abilities often observed in preterm children.

## Author Contributions

ML, LF, UA, and KH designed the study; LF and KH collected the data; ML, LF, and KH analyzed and interpreted the data; ML, LF, and KH drafted the first version of the paper and all authors approved of the final version. All authors agree to be accountable for all aspects of the work in ensuring that questions related to the accuracy or integrity of any part of the work are appropriately investigated and resolved.

## Conflict of Interest Statement

The authors declare that the research was conducted in the absence of any commercial or financial relationships that could be construed as a potential conflict of interest.

## Footnotes

1 http://www.panamath.org

2 While not the main focus of these analyses, we also correlated TB children’s ANS acuity and performance on the Arithmetic subtest of the WISC-IV and found no significant correlation, *R* = -0.17, *p* = 0.25. However, the effect size of this relation is similar to previously reported effect sizes (see Chen and Li, 2014, for a meta-analysis) and may be non-significant due to our small sample size here (*n* = 48 TB children after exclusion of three outliers).
